# A Proposed New Strain of Avian Picornavirus in Broiler Chicken from Brazil

**DOI:** 10.1128/genomeA.00012-18

**Published:** 2018-03-22

**Authors:** Ceyla Maria Oeiras Castro, Elaine Hellen Nunes Chagas, Delana Andreza Melo Bezerra, Aline Farias Ribeiro, Sandro Patroca da Silva, Ana Cecília Ribeiro Cruz, Edivaldo Costa Sousa Júnior, René Ribeiro Silva, Joana D’Arc Pereira Mascarenhas

**Affiliations:** a Section of Virology, Evandro Chagas Institute, Ministry of Health, Ananindeua, Brazil; b Institute of Biological Sciences, Federal University of Pará, Belém, Brazil; c Section of Arbovirology and Hemorrhagic Fevers, Evandro Chagas Institute, Ministry of Health, Ananindeua, Brazil

## Abstract

A new strain of avian picornavirus was identified in fecal samples from broiler chickens in a commercial farm in the municipality of Benevides, Pará, Brazil. Genomic analysis showed it to have a nucleotide identity of 78.4% with the family Picornaviridae, genus Avisivirus, and species Avisivirus A, suggesting that this is a possible new strain within this species.

## GENOME ANNOUNCEMENT

The poultry sector in Brazil grows every year, but it can still experience significant economic losses owing to the circulation of viral pathogens (1). Birds are important reservoirs of pathogens, including picornaviruses, that have a worldwide distribution and infect vertebrates of all classes.

The Picornaviridae family is composed of 35 genera and 80 species recognized by the International Committee on Taxonomy of Viruses, including more than 500 types. These viruses are nonenveloped with an icosahedral capsid and a diameter of 30 nm, have single-stranded RNA genomes of positive polarity, and can vary in size from 6.5 kb to 10.1 kb. Their genomic organization contains a single polyprotein, flanked by two untranslated regions, that when cleaved subsequently generated several other proteins (2).

Chickens are important sources of food, and thus their being affected by gastrointestinal infections negatively impacts the production of meat and eggs, a matter of concern for both animal and human health (3, 4). There is currently a lack of information about the diversity of picornaviruses in birds, with avian encephalomyelitis virus being the only well-studied picornavirus found in chickens in recent years (3). In this study, a pooled fecal sample was collected in July 2009 from 41-day-old broiler chickens of the genus Gallus from a commercial farm located in the municipality of Benevides in the state of Pará, Brazil.

DNA sequencing was performed using a read synthesis system. The cDNA library was prepared and sequenced on an Illumina MiniSeq platform using the methodology described in the Nextera XT DNA library preparation kit (5). The sequencing generated 2,761,286 reads, and the genome was assembled using a hybrid methodology of *de novo* assembly and reference mapping with the IDBA-UD (6) and Geneious version 8.1.9 (7) programs, respectively. Of the 9,737 contigs generated, 66 were related to members of the family Picornaviridae and genus Avisivirus. The assembly by reference mapping showed that 1,675 reads generated a single unitig related to strain Pf-CHK1-AsV (GenBank accession no. KT880669), demonstrating 78.4% nucleotide identity with this genome, with polyprotein regions P1, P2, and P3 showing 76.2%, 85.7%, and 86.4% nucleotide identities and 89%, 99.4%, and 99% amino acid identities, respectively. The average coverage was 32.5×, and the GC content was 45.5%. The genome was annotated and curated using a database containing 129 genomes belonging to the Picornaviridae family. After sequence editing, the genome showed a final length of 7,342 bp.

These results strongly suggest that this strain (AVE052/AsV) is new and belongs to the family Picornaviridae, genus Avisivirus, and species Avisivirus A, emphasizing the importance of picornavirus monitoring in chickens and other poultry by metagenomic analysis to discover new pathogens that may negatively impact poultry and egg production and cause veterinary health concerns.

### Accession number(s).

The whole-genome sequence reported here has been deposited in GenBank under the accession no. MG708279.
